# Identification of a novel DNA repair inhibitor using an *in silico* driven approach shows effective combinatorial activity with genotoxic agents against multidrug‐resistant *Escherichia coli*

**DOI:** 10.1002/pro.4948

**Published:** 2024-03-19

**Authors:** Lorenzo Bernacchia, Antoine Paris, Arya Gupta, Robert J. Charman, Jake McGreig, Mark N. Wass, Neil M. Kad

**Affiliations:** ^1^ School of Biological Sciences University of Kent Canterbury UK

**Keywords:** antimicrobial, Antibiotic resistance, cancer chemotherapy, cooperativity, inhibitor, Microbial infection, Nucleotide excision repair, protein inhibition, virtual screen

## Abstract

Increasing antimicrobial drug resistance represents a global existential threat. Infection is a particular problem in immunocompromised individuals, such as patients undergoing cancer chemotherapy, due to the targeting of rapidly dividing cells by antineoplastic agents. We recently developed a strategy that targets bacterial nucleotide excision DNA repair (NER) to identify compounds that act as antimicrobial sensitizers specific for patients undergoing cancer chemotherapy. Building on this, we performed a virtual drug screening of a ~120,000 compound library against the key NER protein UvrA. From this, numerous target compounds were identified and of those a candidate compound, Bemcentinib (R428), showed a strong affinity toward UvrA. This NER protein possesses four ATPase sites in its dimeric state, and we found that Bemcentinib could inhibit UvrA's ATPase activity by ~90% and also impair its ability to bind DNA. As a result, Bemcentinib strongly diminishes NER's ability to repair DNA in vitro. To provide a measure of in vivo activity we discovered that the growth of *Escherichia coli* MG1655 was significantly inhibited when Bemcentinib was combined with the DNA damaging agent 4‐NQO, which is analogous to UV. Using the clinically relevant DNA‐damaging antineoplastic cisplatin in combination with Bemcentinib against the urological sepsis‐causing *E. coli* strain EC958 caused complete growth inhibition. This study offers a novel approach for the potential development of new compounds for use as adjuvants in antineoplastic therapy.

## 1 INTRODUCTION

De novo drug discovery requires significant investment of time and funds with no insurance that an effective drug will be derived (Schlander et al., 2021). This is especially true of antibiotics, where the returns on decades of investments are not offset by financial gains of their sales leading to major pharmaceutical companies abandoning the sector (McKenna, 2020). Furthermore, drugs currently available are rapidly becoming ineffective due to the global spread of antimicrobial drug resistance (AMR). One‐third of the reported *Escherichia coli* bloodstream infections are caused by drug‐resistant bacteria, which are challenging to treat, resulting in an increased severity and mortality (*Global Antimicrobial Resistance and Use Surveillance System (GLASS) report: 2021*, 2021). In England, cases of infection are steadily increasing over time; between 2020 and 2021, there were reported almost 40,000 *E. coli* bacteraemia cases, more than 40% as urinary tract infections (UTI) and nearly 20% were hospital‐onset cases, with 6000 people dying within 30 days of contracting the disease (Public Health England, 2021). Furthermore, new antibiotic resistant strains are constantly forming and isolated contributing to the loss of effective therapeutic options (J. Zhu et al., 2023), These facts are particularly concerning for cancer patients who often suffer from severe neutropenia induced by antineoplastic agents compromising the immune system (Crawford et al., 2004). Combined with enhanced pathogen penetration due to the destruction of physical barriers, tumoral expansion, and surgical procedures (Rolston, 2017; Zembower, 2014), infections are often associated with death in cancer patients (Nanayakkara et al., 2021; Zembower, 2014).

In addition to damaging the DNA of cancerous and host cells, certain anticancer agents damage the genomes of bacterial cells. Defending the bacterial genome are numerous repair pathways, among which a key player is Nucleotide Excision Repair (NER). This ATP‐dependent, multiprotein DNA repair system is mechanistically conserved between eukaryotes and prokaryotes, but structurally divergent (Petit & Sancar, 1999). In bacteria, NER primarily repairs UV damage, but can also repair a wide range of DNA‐distorting lesions, including those caused by alkylating agents such as cisplatin (Truglio et al., 2006). NER is initiated by UvrA which binds to DNA to locate the damage. Once UvrA locates a DNA lesion, it initiates a cascade of events with the other members of the pathway (UvrB, UvrC, and UvrD), culminating in the removal and the resynthesis of the damaged oligonucleotide (Kad & Van Houten, 2012). Monomeric UvrA (UvrA normally exists as a homodimer) possesses two distinct ATPase sites (distal and proximal) connected by a hollow channel that runs under the DNA binding domain (Barnett & Kad, 2019). These ATPase sites work cooperatively (Barnett & Kad, 2019; Case et al., 2019; Kraithong et al., 2021); the distal ATPase is required for checking for DNA lesions, activating the proximal ATPase if damage is detected, which recruits UvrB (Kad et al., 2010; Myles & Sancar, 1991; Stracy et al., 2016). We have previously shown how the inhibition of these ATPases could represent a target for an adjuvant antimicrobial that stalls replication in conjunction with a DNA‐damaging agent (Bernacchia et al., 2022) and how this could be beneficial in therapy for the treatment of co‐infections in cancer patients (Bernacchia et al., 2023).

In this study, we deployed two strategies to facilitate the discovery process: computational aided drug design and drug repurposing (Ashburn & Thor, 2004; Tiwari & Singh, 2022). To broaden the search while avoiding the possibility of finding unavailable or difficult‐to‐synthesize compounds, we established an easy‐to‐use pipeline for virtual screening to evaluate ~120,000 diverse drug‐like molecules already reported active in vitro. These compounds were docked against a computationally generated UvrA structure (AF‐P0A698‐F1) (Jumper et al., 2021; Varadi et al., 2022), to generate numerous potential leads. We selected one top hit (Bemcentinib) and found it was able to inhibit UvrA's ATPase, and using single molecule imaging, we also showed the compound prevents DNA binding, confirming the in silico results directly. These in vitro results indicate potential disruption of the whole pathway. To confirm this, we assessed Bemcentinib's ability to disrupt NER incision with two different in vitro incision assays. Furthermore, we assessed its ability to impair bacterial growth in vivo using an efflux deficient strain (∆*tolC*) and a reference DNA damaging agent (4‐nitroquinoline 1‐oxide, 4‐NQO; Bernacchia et al., 2022). Having shown the potential of this drug for combinatorial therapy, we suggest a real‐world application by demonstrating the compound's ability to sensitize bacteria to the DNA‐damaging agent cisplatin in the clinical isolate *E. coli* ST131 EC958, responsible for serious multi‐drug infections (Forde et al., 2014; Johnson et al., 2010; Lau et al., 2008; Nicolas‐Chanoine et al., 2008; Paitan, 2018). Altogether, the results represent the first steps toward the identification of a new compound for the treatment of infections associated with DNA damaging chemotherapeutics that cause neutropenia.

## 2 RESULTS

### 2.1 In silico screening identifies several possible NER inhibitors

Nucleotide excision repair represents an attractive and underexplored target because of its role in DNA repair following damage induced by several agents, including cancer chemotherapeutics. UvrA lies at the beginning of the pathway, and its deletion impacts bacterial survivability when exposed to genotoxic agents (Bernacchia et al., 2022). Therefore, we implemented an in silico screening approach that considered a computationally generated structure of UvrA (AlphaFold) as rigid and probed a broad search space, including both ATPase pockets and the channel connecting them. We docked ~120,000 unique compounds from a library of chemicals reported active in vitro using AutoDock Vina. This approach utilized multiple cores to increase the docking speed (The Scripps Research Institute, 2020); however, depending on the molecule docked, an increase in the number of CPUs allocated for that task did not represent a significant improvement. Therefore, submitting different molecules and allocating multiple jobs per CPU was a more effective approach to reduce the screening time. We found that with the eight cores available in our system, allocating up to 32 jobs resulted in a ~40% decrease in the total screening time (Figure S1).

**FIGURE 1:**
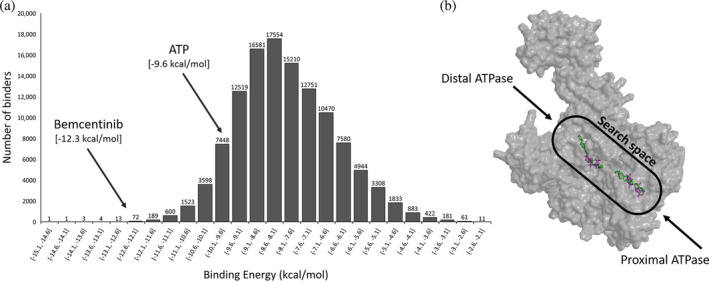
In silico screening of UvrA. (a): Histogram showing the distribution of binding affinities from the compound screen. Both ATP (−9.6 kcal/mol) as a reference and Bemcentinib (−12.3 kcal/mol) are shown. (b): Structure of monomeric UvrA (AlphaFold) showing the search space used for the virtual screening and the docking conformations of ATP (magenta) and Bemcentinib (green) from the models produced by AutoDock Vina.

After a docking analysis, AutoDock Vina outputs the computed binding energies of the screened compounds. The resulting binding energies were plotted as a histogram (Figure 1a). Using the ATPase site as a reference (Figure 1b), we manually inspected the first 50 compounds in the list searching for promising characteristics and accessibility. Among these shortlisted compounds, we found phosphate molecules such as cGAMP, Cyclic di‐AMP, Myo‐inositol trispyrophosphate, and low specificity compounds like tetracosafluorophenanthrene. Furthermore, two of the hits obtained *N*‐(9‐Fluorenylmethoxycarbonyl) Doxorubicin and Hinokiflavone were analogues of compounds already highlighted as possible NER inhibitors in our previous study (Pirarubicin and Apigenin) (Bernacchia, 2023; Bernacchia et al., 2023). Due to its availability and promising characteristics, we progressed Bemcentinib for in vitro evaluation.

### 2.2 Bemcentinib is an effective antagonist of UvrA's ATPase activity in vitro

**FIGURE 2:**
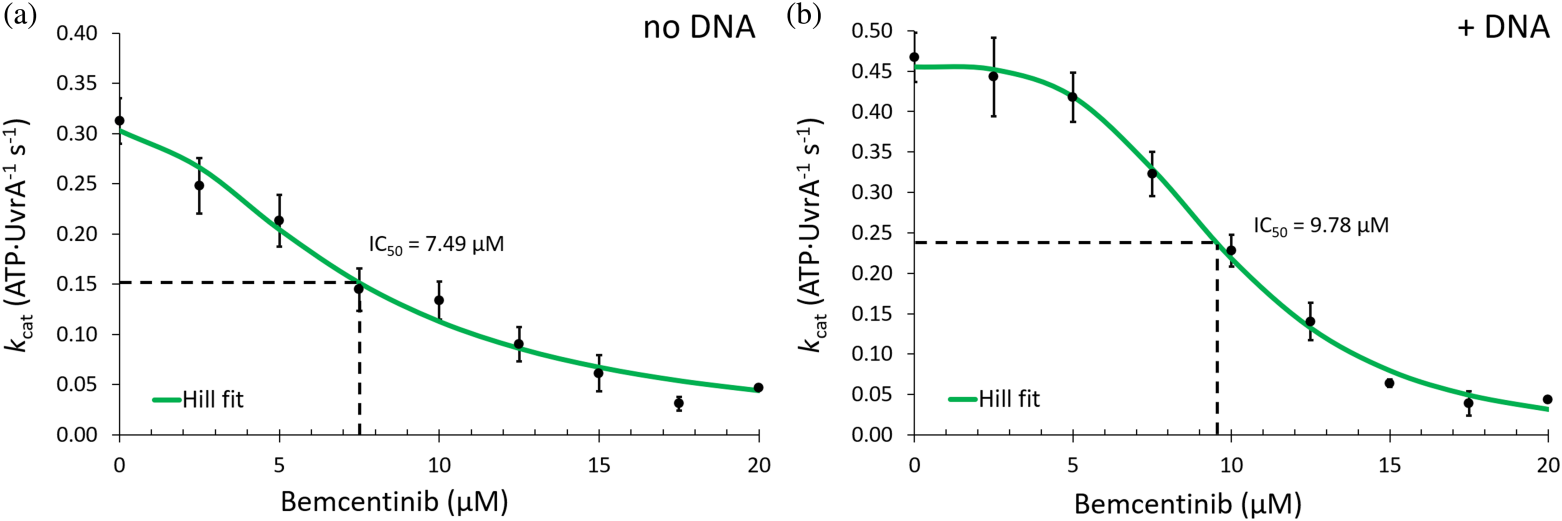
Bemcentinib inhibits UvrA's ATPase activity. A titration of Bemcentinib was and the ATPase activity was measured. (a): ATPase rates measured in the absence of DNA, the green line represents a Hill curve fitted to the data (see Section 4) revealing a ~1.8 Hill coefficient and IC_50_ of 7.49 μM. (b): In the presence of pUC18 DNA the ATPase rate visibly appears more cooperative, leading to a fitted Hill coefficient of ~3.6 and IC_50_ of 9.78 μM. The experiments were repeated three times. The error bars represent the standard error of the mean (*n* ≥ 3).

To validate the virtual screening results, we tested Bemcentinib's ability to inhibit purified recombinant UvrA's ATPase activity in vitro using an NADH‐linked ATPase assay in the presence and absence of DNA. UvrA's homodimeric complex has four ATP binding sites that communicate (Barnett & Kad, 2019; Case et al., 2019; Kraithong et al., 2021). Figure 2 shows the reduction in *k*_cat_ for ATP when UvrA was titrated with the inhibitor in the presence and absence of pUC18 DNA. Both inhibition curves fit well to a Hill relationship, allowing for the estimation of the degree of cooperativity among the ATPase sites. In the absence of DNA (Figure 2a), the fit provides a IC_50_ of 7.49 (± 0.69) μM with a Hill coefficient of ~1.8, indicating positive cooperativity between two possible sites, consistent with a previous study of ADP inhibition (Myles et al., 1991). Interestingly, when DNA was added to the solution to stimulate the ATPase activity (Barnett & Kad, 2019; Bernacchia et al., 2022), we observed a marked increase in cooperativity. The calculated Hill coefficient was ~3.6 with an IC_50_ for the compound to 9.78 (± 0.23) μM, suggesting more ATPase sites were involved (Figure 2b).

### 2.3 Bemcentinib disrupts UvrA's ability to bind to DNA in vitro

**FIGURE 3:**
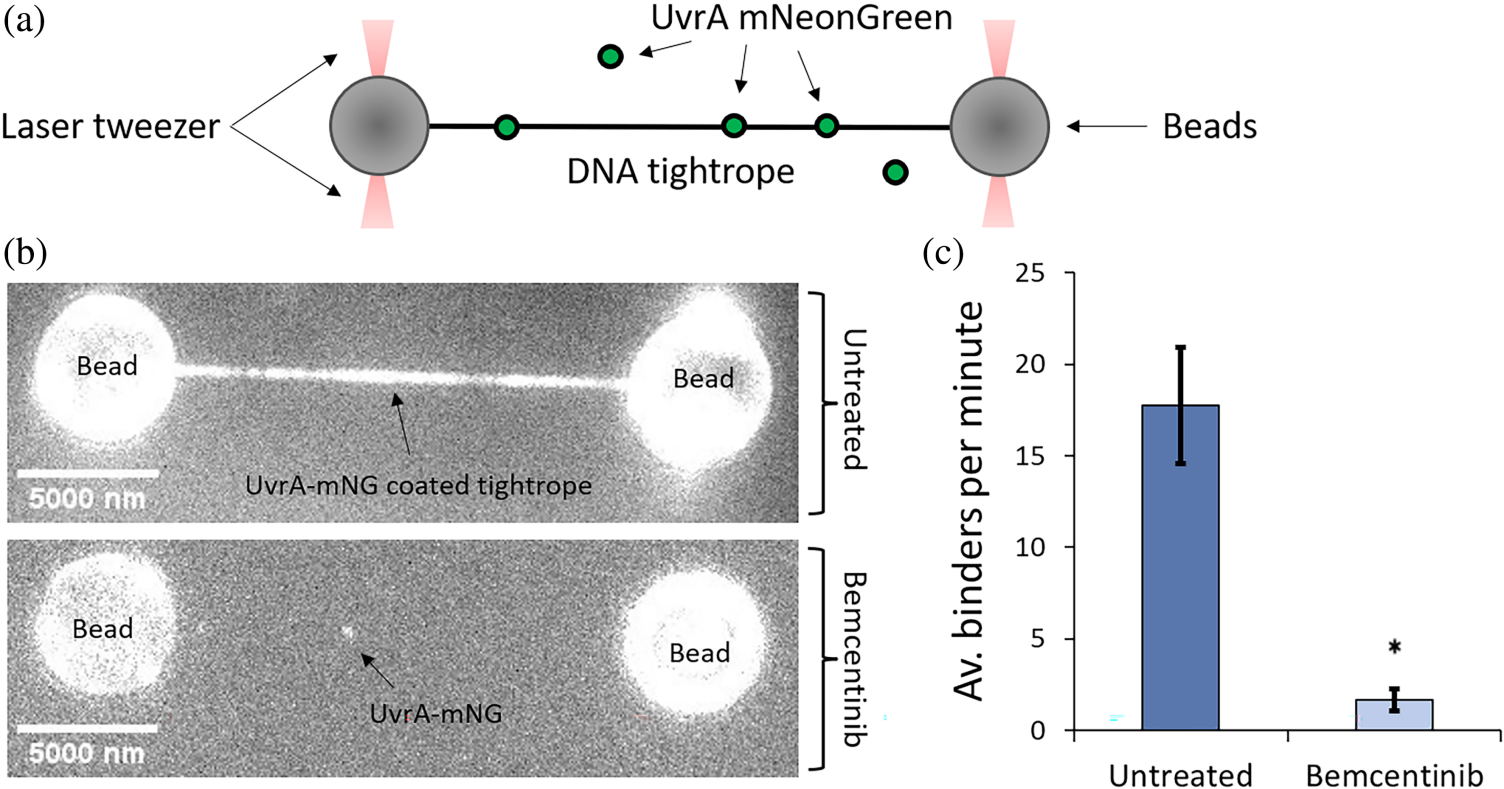
Bemcentinib inhibits UvrA binding to DNA. (a): Schematic of the laser tweezer system. Beads coated in streptavidin are captured within optical traps. Biotinylated lambda DNA is tethered to the beads and put under tension. Fluorescently labeled UvrA is free to bind the unlabeled DNA. (b): DNA strand observed in channels containing UvrA‐mNeonGreen in the absence or presence of 50 μM Bemcentinib (three DNA strands were visualized for each condition). DNA tension was 50 pN and the flow pressure was 0.3 Pa for 5 min prior to imaging. (c): Average binders per minute at 50 pN of tension during 10‐min‐long videos for untreated or treated samples with 20 μM of Bemcentinib (*n* DNA strand = 6 for each condition, *p*‐value ≤0.0006), the error bars represent the standard error of the mean (*n* untreated: 824, *n* treated: 99) and the * indicated statistical significance (*p*‐value ≤0.05).

We have previously shown that a reduction in the ATPase activity affects the ability of UvrA to bind DNA (Barnett & Kad, 2019; Bernacchia et al., 2022; Charman & Kad, 2022). We decided to use the C‐trap optical tweezer system to assess the effect of Bemcentinib on the binding of UvrA‐mNeonGreen to DNA. This system allows the capture of double‐stranded DNA between beads (Figure 3a) and was used to assess DNA binding at the single molecule level with and without Bemcentinib. UvrA C‐terminally tagged with mNeonGreen (UvrA‐mNG) was used to decorate DNA (Bernacchia et al., 2022, 2023) and then challenged with 50 μM Bemcentinib. This significantly decreased UvrA‐mNG binding to the DNA strand (Figure 3b). To quantify the inhibitory effect of Bemcentinib, we reduced its concentration 2.5‐fold to 20 μM to enable enough binding events to be observed to calculate a relative binding affinity. Compared to untreated samples we observed a reduction of ~90% in the number of binders per minute (Figure 3c).

### 2.4 Bemcentinib inhibits NER incision

**FIGURE 4:**
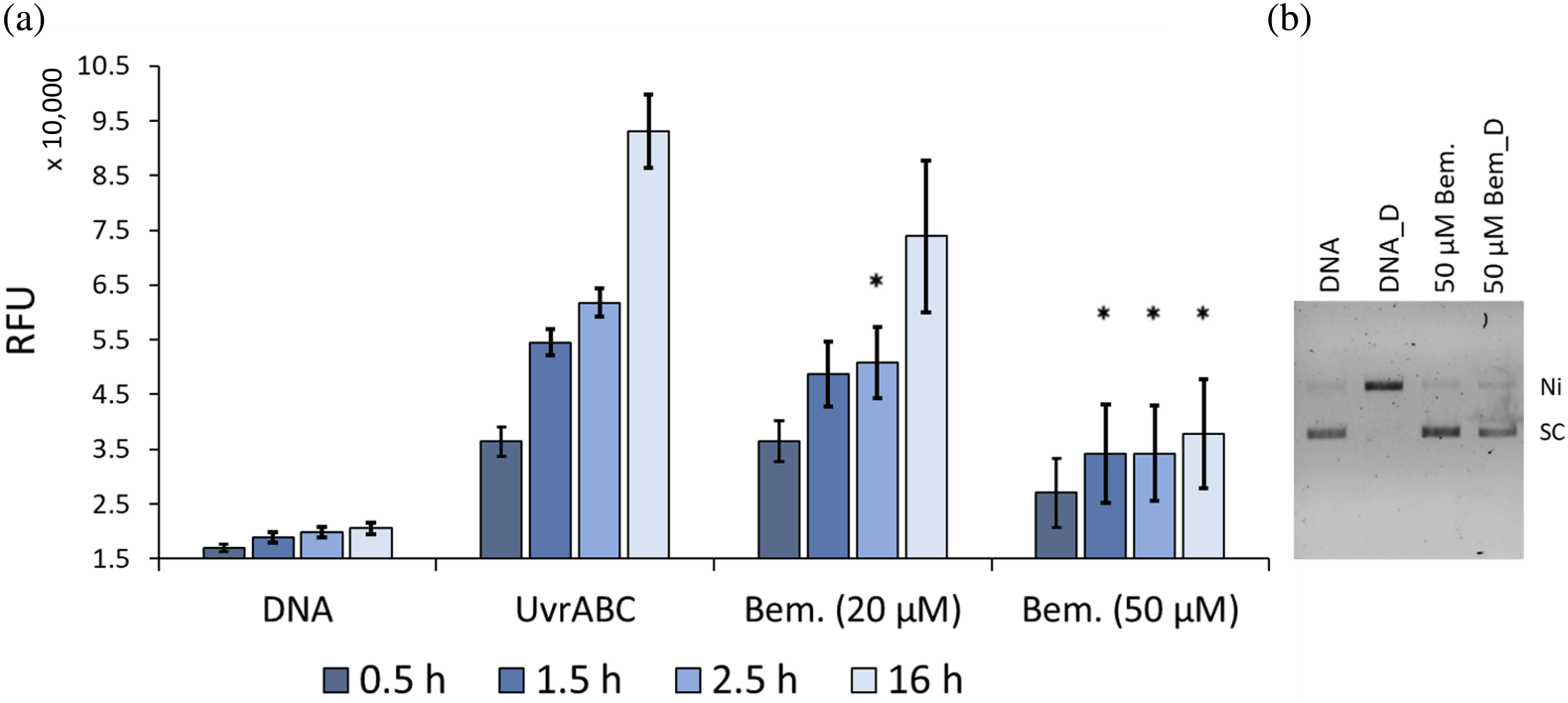
DNA incision by NER is inhibited by Bemcentinib. (a): Bar chart showing the relative fluorescence units (RFU) for the fluorescence‐based incision assay (Bernacchia et al., 2023). Measurements were acquired over time in a plate reader at 37°C. “DNA” indicates the quenched oligonucleotide without any NER enzymes present, providing the background fluorescence signal. “UvrABC” is incision with all NER enzymes present without inhibitor as a positive control. “Bem. (20 μM)” shows that 20 μM of Bemcentinib cannot efficiently inhibit incision. However, 50 μM of Bemcentinib (“Bem. 50 (μM)”) significantly reduces the incision of DNA at 1.5, 2.5, and 16 h compared with untreated “UvrABC.” The fluorescence‐based incisions were repeated three times with two technical replicates, the error bars represent the standard error of the mean (*n* = 6). The *p*‐values are considered significant (*) if the ≤0.05 when compared with the untreated control. (b): Gel‐based incision assay. The gel shows undamaged (DNA) and UV‐damaged (DNA_D) at 37°C in the presence of UvrA, UvrB, UvrC. Incision leads to a loss of super‐coiled DNA (SC) to the nicked band (Ni). In the presence of 50 μM Bemcentinib no loss of super‐coiling is seen, confirming the inhibitory activity of Bemcentinib. This gel is representative of multiple independent measures.

Having confirmed that Bemcentinib inhibits UvrA's ATPase and its ability to bind DNA we evaluated its action against the full NER system in vitro. Briefly, our fluorescence‐based incision assay (Bernacchia et al., 2023) can measure the incision of a fluorescein‐modified oligonucleotide by tagging one strand with a fluorophore and the other with a fluorescence‐quenching probe (Figure 4a). After NER has identified and confirmed the lesion, a sequential incision produces short oligonucleotides that melt at 37°C. The separation of the oligonucleotides de‐quenches the fluorophore resulting in a fluorescence enhancement proportional to the incision. The reactions were monitored in the presence and absence of Bemcentinib for 16 h at 37°C (Figure 4a). The untreated sample (UvrABC) shows the time evolution of the de‐quenched fluorescence. Surprisingly, when the proteins were treated with 20 μM Bemcentinib, no change compared to the untreated control was observed. However, when the concentration of the inhibitor was raised to 50 μM, a significant reduction in incision was measured at 1.5, 2.5, and 16 h with up to 76.1% for the last timepoint observed. To further confirm this result, we measured the shift from supercoiled to open circle DNA caused by incision (Figure 4b). This experiment clearly showed that 50 μM of the compound significantly inhibits incision.

### 2.5 In vivo assessment of Bemcentinib's adjuvant activity when combined with DNA‐damaging agents

**FIGURE 5:**
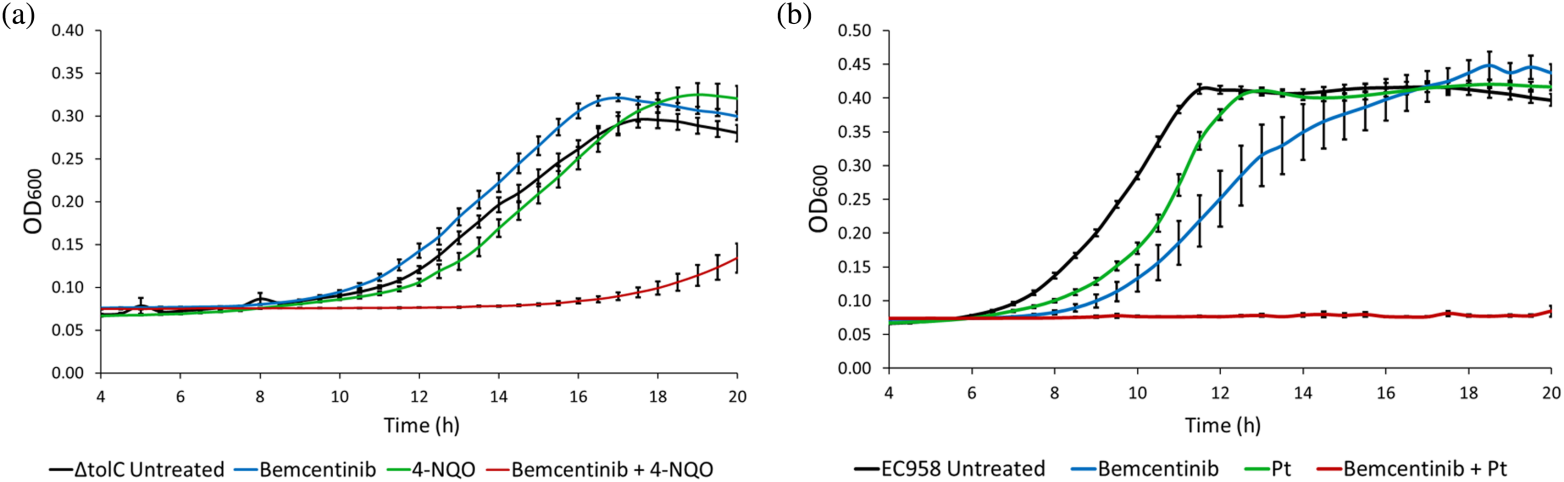
Bemcentinib impairs *E. coli* survival when combined with genotoxic agents. (a): Growth curves acquired in a plate reader show MG1655 ∆tolC in the presence and absence of 1.56 μg/mL Bemcentinib with and without 10 μM 4‐NQO. Both Bemcentinib and 4‐NQO have a negligible effect on growth, but when combined, they cause a significant delay in replication. (b): Growth curves show the combinatorial effect of 25 μg/mL Bemcentinib and 3.125 μg/mL cisplatin (1/4 of its MIC) in the clinical isolate EC958. Both cisplatin and Bemcentinib marginally delay the mid‐exponential in growth but, when combined, show complete inhibition. It is likely the greater concentration required to reach MIC underlies this difference. The growth curves were calculated with multiple technical and biological replicates on independent days. The error bars represent the standard error of the mean (*n* ≥ 6).

After showing that Bemcentinib inhibits NER in vitro, we set out to evaluate its in vivo activity. Firstly, we measured the minimal inhibitory concentration of Bemcentinib in *E. coli* MG1655 *ΔtolC* and *E. coli* MG1655 *ΔtolC ΔuvrA*. MIC values (Figure S2) for either strain were identical at 3.13 μg/mL, indicating that the compound does not generate NER substrates. To detect if the compound can affect bacterial growth in the presence of a DNA‐damaging agent, we used 4‐NQO previously reported to create NER substrates (Bernacchia et al., 2022; Ikenaga et al., 1975; Kondo, 1977). When the *ΔtolC* strain was exposed to either 4‐NQO or Bemcentinib at sub‐MIC concentrations (compare blue to black lines and green to black lines), no critical changes in growth were observed. However, when the two compounds were used together at sub‐MIC concentration, a major delay in cell division was measured, leading to minimal growth after 20 hours of observation (Figure 5a; red vs. black lines).

To bring our results closer to the clinic, we investigated the inhibitory effects of Bemcentinib on a multi‐drug‐resistant *E. coli* strain (EC958). Furthermore, we used cisplatin, which is known to create NER substrates and is already used in cancer chemotherapy (Dasari & Bernard Tchounwou, 2014; Husain et al., 1985). Bemcentinib showed an increased MIC in the clinical strain (50 μg/mL) when compared to the wild‐type MG1655 (25 μg/mL) (Figure S2). We have previously shown the MIC of cisplatin for EC958 (Bernacchia et al., 2023), is the same between the clinical and the wild type strains (12.5 μg/mL) (Gupta et al., 2022). Combining these drugs had a significant effect of the growth of EC958 (Figure 5b). When EC958 is treated with sub‐MIC quantities of Bemcentinib alone (25 μg/mL, compare black to blue line) or cisplatin alone (3.125 μg/mL, compare black to green line), only a marginal delay in bacterial growth is seen. However, complete inhibition of growth was recorded when the two agents were combined (compare black to red line).

## 3 DISCUSSION

Advancements in antimicrobial treatments are needed to replace and augment current therapies due to increased bacterial resistance (O'Neill, 2016). Combination therapies are used predominantly in cancer chemotherapy and cardiovascular diseases (Bhatia et al., 2020; Chen & Lahav, 2016; Guerrero‐García & Rubio‐Guerra, 2018), however, such therapies can be also applied to antimicrobial treatments offering opportunities to breathe new life or extend the reach of existing drugs (Evans et al., 2022; Wang et al., 2022).

Here, we have developed a combined drug strategy that enables the exploitation of a newly explored target, UvrA (Bernacchia et al., 2022, 2023) to act as a sensitizer to the antimicrobial effects of the cancer chemotherapeutics. Using in silico screening of a key protein (UvrA) essential for the efficient repair of a wide spectrum of DNA damage types, we have identified a number of compounds with high‐predicted binding affinities. Of these, we selected and studied Bemcentinib by performing a series of biochemical tests that confirm its inhibitory activity in vitro. Remarkably this compound, in combination with the cancer chemotherapeutic cisplatin, is effective in inhibiting the growth of the multi‐drug resistant *E. coli* strain EC958 in the presence of cisplatin. This offers a promising new approach to tackle cancer‐chemotherapy related infections in the future.

### 3.1 Bioinformatics in drug discovery

In recent years, tremendous advancements in computational power and technological accessibility have played a central role in the development of new in silico tools for drug discovery. These include the use of artificial intelligence for protein structure modeling (Jumper et al., 2021), availability of free ultra‐large chemical library repositories (Irwin et al., 2020; Kim et al., 2021) and the development of several accessible tools for bioinformatics (Bragina et al., 2022; Halgren et al., 2004; O'Boyle, Vandermeersch, et al., 2011; Trott & Olson, 2010). As a consequence, fast and inexpensive molecular docking/modeling, binding site prediction and virtual screening is now possible (Tiwari & Singh, 2022), leading to numerous newly designed drugs (Cavasotto et al., 2019; Clark, 2008; Cosconati et al., 2010; Talele et al., 2010).

In this study, we used a Python code that allows for fast virtual screening without demanding hardware requirements based on the use of open‐access AutoDock Vina as the docking algorithm. Our tests show an improved docking speed reducing the total run time by approximately ~40% when multiple jobs were submitted simultaneously. It is important to note that the improvements depend on the number of CPU's allocated for each task, therefore, faster processing will be afforded by systems with more CPUs. Due to the intrinsic approximations and limits of the process (Gimeno et al., 2019; Scior et al., 2012) we avoided the analysis of multiple docking conformations, preventing a potential combinatorial explosion in the number of results. No solution structure of *E. coli* UvrA is available therefore we were able to use an AlphaFold generated model for computational docking. From this, we restricted the time overhead in selecting promising compounds by limiting investigation to the top 50 molecules identified in the screen, when sorted by binding energy. Although there were other potentially interesting compounds, due to limited availability, we progressed Bemcentinib because it possessed a potential use in cancer chemotherapy. In our previous study (Bernacchia et al., 2022), we used in silico docking of an inhibitor discovered using manual screening. Comparing the approaches; manually screening ~3000 compounds took ~5 months, versus this in silico approach using an AI‐generated UvrA structure that took ~2 weeks to screen 120,000 compounds. Therefore, based on the simple standpoint of rapid drug discovery, in silico offers a wider‐ranging capture of compounds in a shorter period of time.

Bemcentinib is a tyrosine kinase (AXL) inhibitor is involved in inhibiting tumor proliferation (Hong et al., 2013; Sang et al., 2022; Zhang et al., 2018; C. Zhu et al., 2019). As with other first in class AXL inhibitors, the compound was designed to inhibit the kinase in its active conformation by disrupting its ATPase activity (C. Zhu et al., 2019). These promising compounds, that are still under investigation for anti‐cancer activity (Sang et al., 2022) could also potentially target UvrA. Despite the library screen containing numerous other tyrosine kinase inhibitors, they scored poorly compared with Bemcentinib. The top‐scoring compound after Bemcentinib was amuvatinib in ranked position 8188 with a binding energy of −9.9 kcal/mol (Bemcentinib −12.3 kcal/mol, ATP −9.6 kcal/mol). Further down in the ranking, we found other members of the same family, such as gilteritinib (pos. 13,652, −9.5 kcal/mol), crizotinib (pos. 15,557–9.4 kcal/mol), TP‐0903 (pos. 24,585–9.1 kcal/mol), and sunitinib (46,257–8.4 kcal/mol) (C. Zhu et al., 2019). These results suggest Bemcentinib is UvrA‐specific, however further direct investigations would be required, since binding energy in computational screens does not provide a full picture of binding.

### 3.2 Bemcentinib impairs NER both in vitro and in vivo

To understand how Bemcentinib inhibits the multi‐site ATPase UvrA we titrated Bemcentinib and measured the ATPase, fitting the results to a Hill curve (Holford & Sheiner, 1981) for multi‐site inhibition. We found Bemcentinib could reduce the ATPase of purified recombinant UvrA up to ~90% with a good IC_50_ in the μM range (~7.5–10 μM ± DNA). The titrations were sigmoidal and fitted well with a Hill curve providing a Hill coefficient of ~1.8 which increased to ~3.6 upon the addition of pUC18 DNA, implying strong cooperativity between the multiple ATP binding sites (Cliff et al., 1999; Holford & Sheiner, 1981; Stefan & Le Novère, 2013). Hill coefficients have been widely used to indicate the number of sites involved, exemplified by the hemoglobin‐oxygen binding curve that possesses a near four‐value for its coefficient (Holford & Sheiner, 1981). Although this approach has limitations (Holford & Sheiner, 1981), it provides the starting point to speculate on the two different inhibition profiles when Bemcentinib is in the presence or absence of DNA.

**FIGURE 6:**
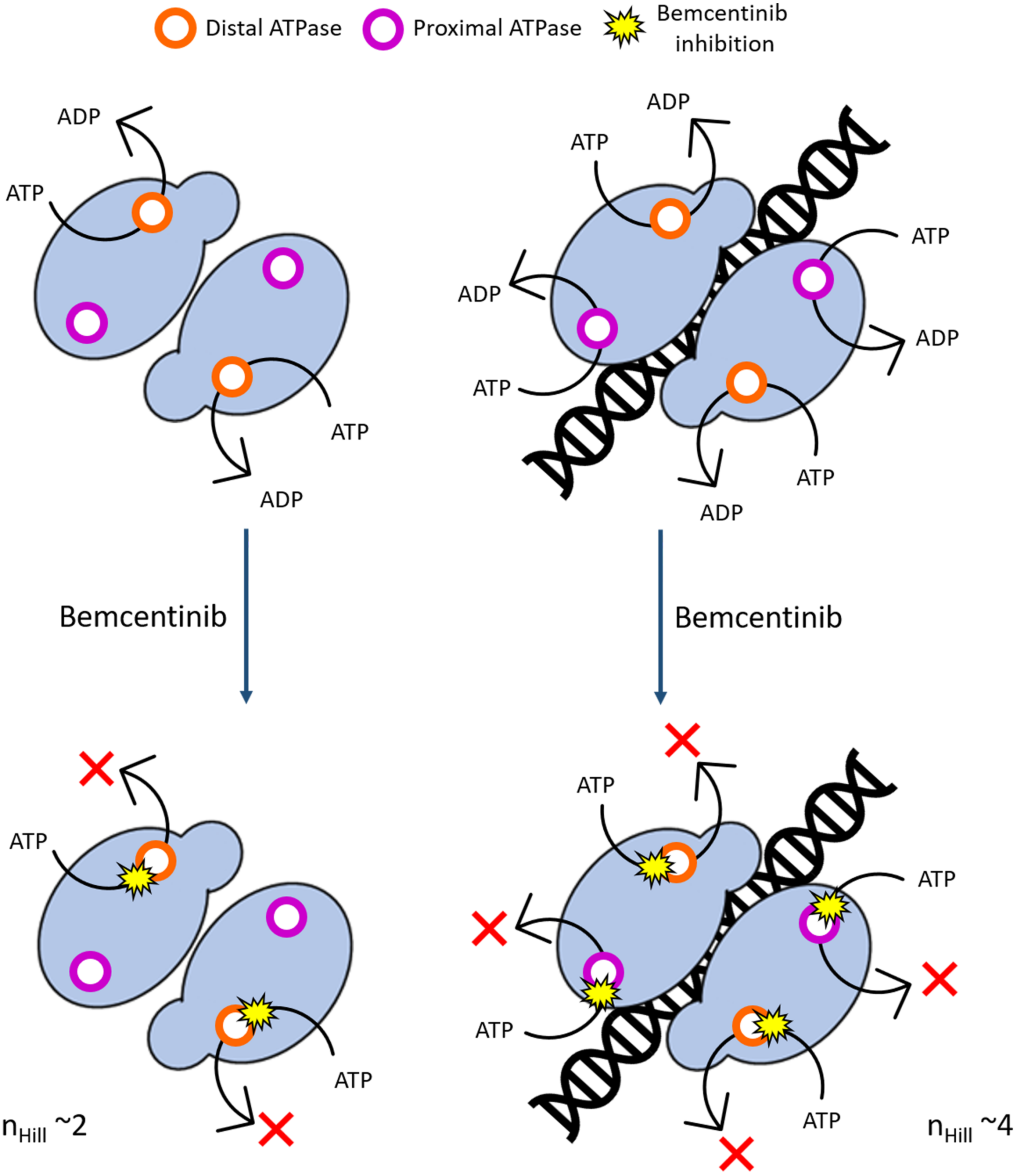
Potential mechanism of UvrA's ATPase inhibition by Bemcentinib. In the absence of DNA (left) only the distal sites (orange) are responsible for the ATP turnover, the inhibition therefore has a Hill coefficient of ~2. With DNA present (right), the proximal sites (purple) contribute to the total ATPase activity, doubling the number of active sites. Bemcentinib is now able to inhibit all of these sites cooperatively resulting the Hill coefficient to ~4.

The homodimer of UvrA possesses four distinct ATP binding sites operating in concert to facilitate DNA binding and UvrB recruitment (Barnett & Kad, 2019; Case et al., 2019; Jaciuk et al., 2011; Kraithong et al., 2021; Myles et al., 1991). Importantly, it has recently shown that the distal ATPase site on UvrA hydrolyses ATP quickly whereas the proximal site has little activity in the absence of DNA (Case et al., 2019). However, when DNA is introduced, both the distal sites and the proximal sites get activated, resulting in a stimulation of the protein catalytic activity through a complex system influenced by structural modifications correlated to DNA binding (Barnett & Kad, 2019; Case et al., 2019; Kraithong et al., 2017). The strong cooperative effects, and their differences in the presence of DNA argue that the distal sites are responsible for the ATP turnover in the absence of DNA, resulting in a principal effect on two sites and a Hill coefficient of ~2. However, upon introducing DNA, the two proximal sites additionally contribute to the ATPase activity raising Hill coefficient to 4. A schematic representation of this mechanism is presented in Figure 6.

Having confirmed the in silico results activity in vitro, we set out to test its ability to disrupt DNA binding by directly observing purified UvrA binding tethered DNA. In agreement with the previously collected data, we measured a ~90% inhibition in DNA binding ability in the presence of 20 μM Bemcentinib. Furthermore, 20 μM Bemcentinib reduced the UvrA lifetime on undamaged DNA by ~40% (Figure S3), implying that the majority of the effect occurs through a reduced attachment rate.

After demonstrating the compound action against UvrA, we investigated its ability to prevent incision when in the presence of the entire complex. We found that 20 μM Bemcentinib could not effectively impair NER‐mediated incision. Although Bemcentinib has IC_50_ of ~10 μM (Figure 2) UvrA has been shown previously to catalytically load UvrB onto lesions (Orren & Sancar, 1989), therefore, even a few active molecules would allow the reaction to proceed in vitro. However, when the concentration was raised to 50 μM, inhibition was much stronger supporting this hypothesis.

Having fully demonstrated Bemcentinib's ability to inhibit NER in vitro, we evaluate its effect on *E. coli*. Using the well‐established NER activator 4‐NQO (Bharati et al., 2022; Ikenaga et al., 1975; Kondo, 1977) with Bemcentinib at sub‐inhibitory concentrations for both compounds, we were able to measure significant growth inhibition of *E. coli* MG1655 *ΔtolC* growth. To demonstrate a possible real‐life application following on from our recent study (Bernacchia et al., 2023). We used the anticancer drug cisplatin (Dasari & Bernard Tchounwou, 2014), which causes DNA adducts that need to be repaired by bacterial NER (Beck et al., 1985; Popoff et al., 1987). Moreover, we extended the testing on a clinically relevant strain, the multidrug‐resistant *E. coli* ST131 (EC958), which is globally responsible for urinary infections (Totsika et al., 2011). When Bemcentinib was incubated below its MIC with a nontoxic concentration of cisplatin (1/4th its MIC (Bernacchia et al., 2023)) I the multidrug‐resistant strain did not grow.

Additional investigations and clinical testing will be necessary to bring this compound to clinical use, despite this our findings suggest that Bemcentinib could be extremely valuable as a cisplatin sensitizer to target bacterial growth in patients receiving this type of anti‐cancer therapy, or as a backbone for further chemical alteration to improve compatibility with the target UvrA.

### 3.3 Bemcentinib's possible role in therapy

One of the most severe problems for cancer chemotherapy patients are infections making them the second leading cause of death among cancer patients (Nanayakkara et al., 2021; Zembower, 2014). Moreover, the rise of multi‐drug resistant bacteria renders ineffective prophylactic antibiotic therapy (Teillant et al., 2015). It is in this area that the data presented in this study suggests that Bemcentinib could offer most hope. We chose to study this compound in combination with cisplatin against a clinically relevant strain found in urinary tract infections (UTIs) and bloodstream infections worldwide (Totsika et al., 2011). Among other cancers, cisplatin is used to treat bladder malignancies, and the compound is excreted in the urine, where it tends to accumulate to high concentrations after administration (Galsky et al., 2012; Safirstein et al., 1984). This means that co‐treatment with Bemcentinib could potentially offer a viable strategy for preventing or treating UTIs, minimizing the need for additional antibiotics.

The advantages of using AXL inhibitors goes further since it has been recently shown that AXL inhibitors enhance chemosensitivity for cisplatin in different cancer types, likely by reducing drug resistance (Hong et al., 2013; Tian et al., 2021). These inhibitors are being studied for lung cancer in which AXL tends to be overexpressed (Sang et al., 2022; Zhang et al., 2018), a disease mainly treated with platinum‐based compounds (Abrams et al., 2003). Bemcentinib was optimized to specifically target the human protein AXL (Zhu et al., 2019), therefore, the anticancer/Bemcentinib combination could be simultaneously beneficial for infection and improving cisplatin's effects on cancerous cells.

In conclusion, we have undertaken an in silico approach using a computationally generated protein structure used for the virtual screening, to identify an effective molecule against purified UvrA which compromises bacterial nucleotide excision repair. Treatment of bacteria with the lead compound Bemcentinib sensitizes cells to DNA‐damaging agents, specifically cisplatin, even in a multidrug‐resistant strain responsible for globally disseminated infections (EC958). We believe these results demonstrate how Bemcentinib represents a promising candidate both as it is and as a template for further improvements for a new class of antimicrobial molecules to be used in combination with anticancer drugs.

## 4 MATERIALS AND METHODS

### 4.1 Bacterial strains, media, and compounds

We used the reference strains *E. coli* MG1655, *E. coli* MG1655 *ΔtolC*, *E. coli* MG1655 *ΔtolC ΔuvrA*, BL21 *ΔuvrA ΔuvrB*, and the clinical isolate *E. coli* ST131 EC958. The mutant genes were prepared via P1 transduction using the Keio collection (Baba et al., 2006; Bernacchia et al., 2022). Bacteria were grown overnight before the assay in LB Broth, Miller (ThermoFisher), then inoculated in MOPS minimal medium supplemented with 0.4% glucose (Neidhardt et al., 1974). 4‐NQO (Merck) and Bemcentinib (R428) (MedChemExpress) were dissolved in 100% DMSO and stored at −80°C. Cisplatin (Merck) was dissolved in 0.9% w/v in saline and stored at 4°C protected from light.

### 4.2 Protein purification

The plasmids used in this study for recombinant UvrA, UvrB, UvrC, and UvrA‐mNeonGreen were designed as previously described (Bernacchia et al., 2023). Bacteria were grown in selective media (LB Miller) to mid‐exponential (OD_600_) with aeration (at 180 RPM) at 37°C. 0.5 mM IPTG was added to the suspension along with fresh antibiotic and re‐incubated at 18°C overnight. The culture was then pelleted and resuspended in lysis buffer containing 50 mM NaPO_4_ (pH 7.5), 20 mM imidazole, 500 mM NaCl, 1 mM phenylmethylsulfonyl fluoride (PMSF), and a protease inhibitor cocktail ((no EDTA) Thermo Fisher Scientific). The cells were lysed with 100 μg/mL lysozyme and additional sonication. 50 μg/mL DNAse I (Roche) was added to the solution for 30 min just before centrifugation at 20,000 RPM at 4°C. The proteins in the supernatant were then purified using a Proteus “1‐step batch” midi plus spin column (Protein Ark column) containing a Ni‐NTA resin (Thermo Scientific™ HisPur™) equilibrated with 50 mM Na_3_PO_4_ (pH 7.5 for UvrA and B and pH 8 for UvrC), 20 mM imidazole, 500 mM NaCl. Finally, the proteins were eluted with increasing concentrations of imidazole, buffered exchanged in storage buffer (50 mM Tris (pH 7.5)), 500/100/400 mM KCl (respectively for UvrA, UvrB, UvrC), 0.1 mM EDTA, 2.5/2.5/5 mM DTT (respectively for UvrA, B and C), 50% v/v glycerol and stored at −20°C. The concentrations were estimated by reading the absorbance at OD_280_. The lysate containing UvrA‐mNeonGreen for the optical trapping experiment was prepared as previously described (Bernacchia et al., 2023).

### 4.3 In silico screening

For the in silico work, we used the open‐access software AutoDock Vina, OpenBabel, and AutoDock tools 1.5.7 (Forli et al., 2016; O'Boyle, Banck, et al., 2011; Trott & Olson, 2010). Due to the lack of an available *E. coli* crystal structure, the protein structure (UvrA: AF‐P0A698‐F1) was retrieved from the AlphaFold Protein Structure Database (Jumper et al., 2021; Varadi et al., 2022). This was chosen due to its close alignment to the Geobacillus structure (PDB: 3UX8). A collection of drugs tested active in vitro and available for sale was retrieved from ZINC15 (173,234 entries, among which 117,760 were unique) (Sterling & Irwin, 2015) and converted in bulk into .pdbqt format using OpenBabel GUI. The protein structure was prepared by the addition of polar hydrogens and conversion in .pdbqt format using AutoDock Tools 1.5.7. ATP was docked into the protein with a maximized search space to verify the ability of the algorithm to find the ATPases cassette. Then, appropriate coordinates were used to define a search space covering the ATPase cassettes and the tunnel connecting them. A Python code was written to allow multiple job submissions simultaneously to increase the speed of the screening and to print the results in a .csv file in ascending order of binding energy for automatic and simplified sorting (available on GitHub, please see data availability statement below). Before performing the screening, a set of 500 molecules and a set of 10,000 molecules were tested, changing the level of parallelization to estimate the fastest conditions. The first 50 unique compounds were evaluated singularly.

### 4.4 NADH‐linked ATPase assay

Determining ATPase rates was performed as previously described (Bernacchia et al., 2023), and in the presence or absence of 0.1 ng/μL pUC18 DNA. Data were acquired from multiple independent measurements, and the errors are reported as the standard error of the mean. The data were fitted in Excel, and the fitting was calculated using Data Solver and SolvStat using the Hill equation:

$$\text{Effect}=k_{\mathrm{cat}\left(0\right)}\left(1-\frac{{\left[I\right]}^{n}}{{\left[{\mathrm{IC}}_{50}\right]}^{n}+{\left[I\right]}^{n}}\right)$$

where *k*_cat(0)_ is the uninhibited *k*_cat_, *I* is the Bemcentinib concentration, *IC*_50_ is the half‐maximal inhibitory concentration for Bemcentinib, and *n* is the Hill coefficient. The equation was adapted from (Holford & Sheiner, 1981).

### 4.5 Single‐molecule imaging

UvrA‐mNeonGreen lysate was prepared as previously described (Bernacchia et al., 2022, 2023), and the concentration was estimated by absorbance at 506 nm. UvrA‐mNeonGreen was used at a concentration of 5 nM in ABC buffer (50 mM Tris pH 7.5, 50 mM KCl, 10 mM MgCl_2_) complemented with 1 mM ATP, 5 mM DTT, and protease inhibitor mix (to a final concentration of 0.07 mg/mL) ((no EDTA) Thermo Fisher Scientific), with or without the addition of 50 or 20 μM of Bemcentinib. To image UvrA‐mNeonGreen interactions with single DNA strands, we used an optical trap system (C‐trap; Lumicks, Netherlands), which allows for the capture of silica beads coated with streptavidin. End‐biotinylated Lambda DNA strands can be tethered between these beads and were used as a substrate for imaging. Imaging was performed at 30% laser power (488 nm), 200 ms exposure at a frame rate of 2 Hz. Exposure to Bemcentinib was performed by moving the DNA strand under 50 pN of tension into a channel with or without Bemcentinib. Flow was applied at 0.3 Pa for 5 min, before the lasers and camera were turned on for imaging. The third frame of the resulting video was used to illustrate the inhibition displayed in Figure 3b. To quantify this effect, the assay was repeated in the absence of flow and videos were recorded for 10 min and analyzed using the TrackMate plugin of ImageJ.

### 4.6 Incision assays

Two methods were used to determine the incision capacity of the system: (1) a fluorescence‐based incision assay and (2) a gel‐based incision assay. Both assays were performed as previously described (Bernacchia et al., 2023). The data were collected in multiple independent measurements, and the error bars represent the standard error of the mean.

### 4.7 Survival assay

Clinical and Laboratory Standards Institute (CLSI) guidelines were adapted to evaluate survivability in different strains as previously described (Bernacchia et al., 2022; Cockerill et al., 2012). Briefly, bacteria were inoculated in fresh LB and grown overnight at 37°C while shaking. Before the experiment, the culture was re‐inoculated in sterile MOPS minimal medium (Neidhardt et al., 1974) supplemented with 0.4% glucose to a final OD_625_ of 0.001 for the assay. The experiments were repeated multiple times with independent measurements. The error bars represent the standard error of the mean, calculated from both the technical and the biological replicates.

## AUTHOR CONTRIBUTIONS

**Lorenzo Bernacchia:** Conceptualization; investigation; writing – original draft; methodology; formal analysis; writing – review and editing. **Antoine Paris:** Methodology; investigation; writing – review and editing; formal analysis. **Arya Gupta:** Investigation; formal analysis. **Robert J. Charman:** Resources. **Jake McGreig:** Resources; software. **Mark N. Wass:** Resources. **Neil M. Kad:** Conceptualization; formal analysis; supervision; project administration; writing – review and editing; writing – original draft; funding acquisition; validation.

## Supporting information


**Data S1.** Supporting Information.

## Data Availability

Additional code is available here: https://github.com/Kad-Lab/Bemcentinib_data.
