# Supplementary material for: Identification of a novel DNA repair inhibitor using an in silico driven approach shows effective combinatorial activity with genotoxic agents against multidrug‐resistant Escherichia coli
Source: Protein Sci. 2024 Mar 19;33(4):e4948. doi: 10.1002/pro.4948 (PMC10949335; doi:10.1002/pro.4948)
Supplement: Supplementary file 1 — Data S1. Supporting Information. [file PRO-33-e4948-s001.pdf]

Time improvements with different levels of parallelization

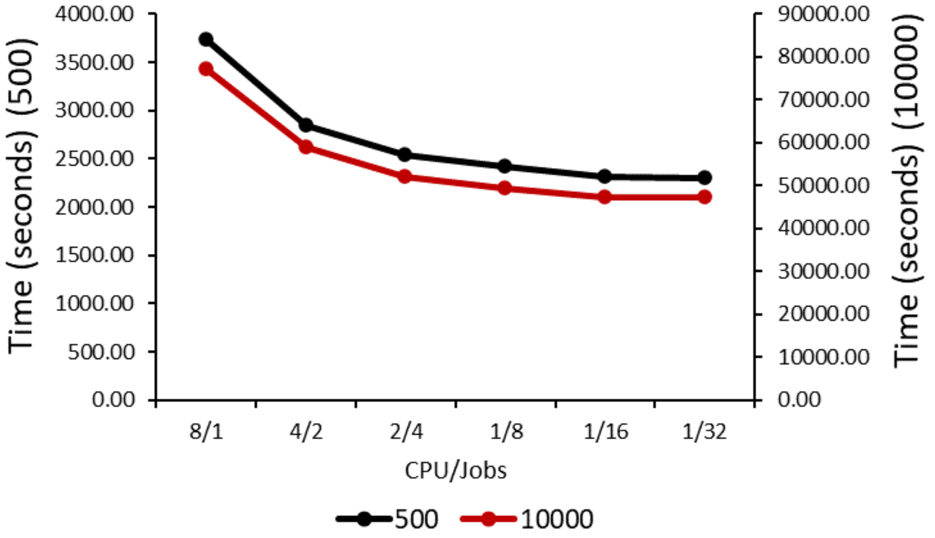

Supplementary figure 1: Two randomly chosen libraries of molecules (respectively 500 and 10000 compounds) were docked using AutoDock Vina. The total time was plotted against the CPU used per job (CPU/jobs). The improvements saturate when the system utilizes 1 CPU per job running a total of 16 simultaneous jobs (1/16). The total improvement in time is ~38%. As can be seen no substantial differences were observed in the saturation point between the two different sized libraries.

Minimal inhibitory concentrations

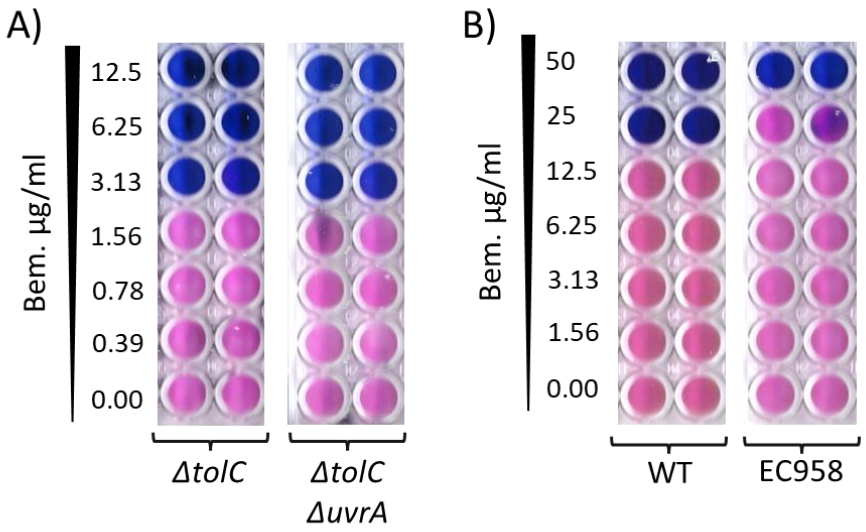

Supplementary figure 2: Bemcentinib MIC in *E. coli*. (**A**) MIC measured after incubation at 37°C for 16 h, followed by a 4 h incubation with resazurin. Bemcentinib shows a MIC of 3.13 µg/ml for normal and the uvrA knockout strain, indicating the compound does not cause damage repaired by NER. (**B**) Bemcentinib has an increased MIC in the wild-type strain MG1655 and in EC958, corresponding respectively to 25 µg/ml and 50 µg/ml, likely due to TolC efflux The pictures are representative images with brightness enhanced (~25%) of 3 biological replicates with 2 technical replicates.

UvrA lifetime on undamaged DNA in the presence and absence of Bemcentinib

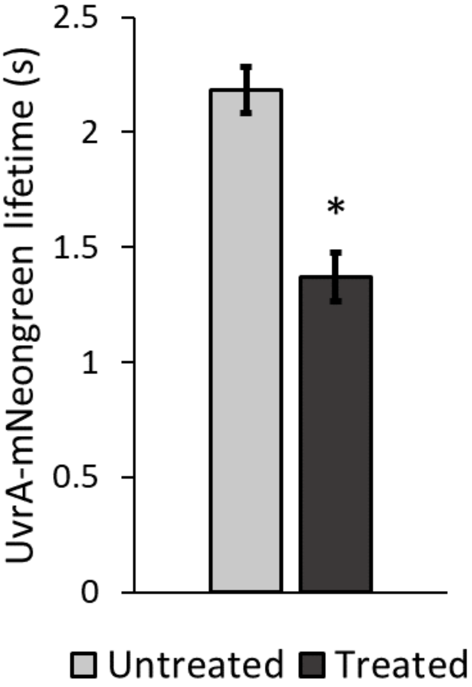

Supplementary figure 3: UvrA lifetimes on undamaged DNA. The lifetime of UvrA-mNeonGreen decreased from 2.18 (± 0.1) s to 1.37 (± 0.11) s when in the presence of 20 µM of Bemcentinib (n= 824 and 99 molecules, respectively; R^2 = 0.98 and 0.94 respectively; P-value = 0.0052).
